# The mediating role of perceived stress between loneliness and medication behavior in Chinese children: a large cross-sectional study

**DOI:** 10.3389/fpubh.2026.1805172

**Published:** 2026-05-22

**Authors:** Lijie Yang, Ming Yang, Yuehang Sun, Yang Yu, Zhongmei Liu, Jing Zhang, Ximu Sun, Han Zhou, Xiaolin Xu

**Affiliations:** 1 Department of Pharmacy, The Sixth Affiliated Hospital of Harbin Medical University, Harbin, China; 2 School of Pharmacy, Daqing Branch of Harbin Medical University, Daqing, China; 3 Department of Pharmacy, Beijing Children's Hospital, Capital Medical University, National Center for Children's Health, Beijing, China

**Keywords:** children, loneliness, mediating effect, medication adherence, perceived stress

## Abstract

**Background:**

Loneliness, as a critical psychosocial factor influencing medication adherence in pediatric populations, warrants particular attention in the long-term management of chronic diseases. Based on data from the “Medication Literacy Investigation of Chinese Children” conducted across children's hospitals in 18 provinces and municipalities in China from October 2023 to May 2024, this study explores the relationship between loneliness and medication management and examines the mediating role of perceived stress in this association.

**Methods:**

A total of 4,404 children and adolescents aged 8–18 years (2,337 girls and 2,067 boys) were included. Data on loneliness, perceived stress, and medication adherence were collected through questionnaires. A mediation effect model was used to analyze the relationships among the variables.

**Results:**

Higher levels of loneliness were significantly associated with lower medication adherence (β = −0.670, *P* < 0.001). Perceived stress partially mediated this relationship, with an indirect effect of β = −0.280 (95% CI: −0.331 to −0.229), accounting for approximately 41.8% of the total effect. This suggests that in pediatric chronic disease management, loneliness not only directly affects medication behavior but also further reduces adherence by exacerbating perceived stress.

**Conclusion:**

As the first study to verify the partial mediating effect of perceived stress in a Chinese pediatric population, it highlights the necessity of addressing both loneliness and stress regulation in interventions aimed at improving medication adherence—particularly in the long-term management of children with chronic conditions—through integrated and multidimensional psychosocial approaches to enhance health management outcomes.

## 1 Introduction

The incidence of chronic diseases has exhibited a consistent annual increase. On a global scale, between 10 and 20% of children are affected by chronic diseases, posing considerable challenges in terms of treatment and care management (1). The adoption of appropriate health behaviors by patients is essential for both the prevention and management of chronic diseases. Among these behaviors, adherence to prescribed medication regimens is vital for achieving therapeutic outcomes in various diseases. However, it is estimated that 30 to 50% of adults with chronic conditions do not adhere to their prescribed medication protocols (2). In the management of certain illnesses, patients and their caregivers are often held accountable for poor medication adherence (3). Additionally, pediatric patients often exhibit suboptimal adherence to medication regimens, which can be attributed to factors such as developmental immaturity (4), limited cognitive capacity, and medication-related anxieties (5, 6). In China, Xin Ni et al. emphasize the importance of prioritizing interventions for pediatric mental health conditions, including depression and anxiety (7). Currently, fluctuations in psychological state are recognized as a critical factor affecting medication adherence (8). Nevertheless, research in this area remains limited, with few studies exploring the mechanisms by which psychological states affect adherence to medication regimens.

Loneliness is defined as the absence of meaningful social connections with others (9). It is a common emotional experience that children may encounter throughout their psychological development (10). The incidence of loneliness and depression among young individuals has risen markedly (11). China's one-child policy has significantly altered family structures, leading to increased time spent alone among children and insufficient family support, which may be associated with greater feelings of loneliness and a higher risk of depressive symptoms (12). The duration of loneliness appears to be a predictor of subsequent mental health issues (13). Pediatric patients often experience restrictions in their daily activities due to disease-related limitations, which may exacerbate feelings of loneliness. This loneliness can adversely affect their mental health and subsequently influence their health-related behaviors. However, research in this area remains limited.

Perceived stress constitutes a complex adaptive response crucial for an organism's survival in threatening situations (14). Over an extended period, stress has been a central focus of research with in the health sciences due to its capacity to influence health outcomes by modifying health-related behaviors (15). In pediatric patients, a variety of factors—including the nature of the disease, the treatment regimen, familial economic strain, academic pressures, and interpersonal relationships—can contribute to their perceived stress. Prolonged or excessive perceived stress may adversely affect not only the mental health of these young patients but also potentially compromise their physical health and treatment outcomes.

In the realm of long-term therapeutic interventions, medication adherence is characterized as the extent to which an individual's behaviors including medication consumption, dietary adherence, and lifestyle modifications—conform to the recommendations collaboratively established with a healthcare professional (16). In pediatric populations, the primary responsibility for ensuring the administration of medication falls to the parents; however, as children mature, this responsibility progressively shifts to the patients themselves (17). This dynamic contrasts with the patterns of medication adherence observed in adult populations, presenting a significant challenge for both patients and parents.

The objective of this study is to evaluate the factors associated with perceived stress in pediatric patients, reveal the possible mediating role of stress between loneliness and medication adherence, provide a mechanism explanation of these factors. This is the first study to explore the relationship between the three in Chinese pediatric patients at the same time. Large samples and standardized scales were used to enhance the representativeness and reliability of the results. Consequently, four hypotheses were formulated: Hypothesis 1 posits that loneliness negatively impacts medication adherence among pediatric patients. Hypothesis 2 suggests that loneliness is a predictor of perceived stress. Hypothesis 3 proposes that perceived stress as a negatively predicts of medication adherence. Hypothesis 4 asserts that perceived stress functions as the principal mediating mechanism connecting loneliness and medication adherence, functioning as an intermediary in this relationship.

## 2 Method

### 2.1 Study design

The research utilized data from Medication Literacy Investigation of Chinese Children (MLICC), a comprehensive, cross-sectional survey from October 2023 to May 2024, selecting children's hospitals from 18 provinces and cities in China (excluding Hong Kong, Macau, and Taiwan). This research was conducted following approval by the Ethics Committee of Beijing Children's Hospital Affiliated to Capital Medical University (Ethics Approval Number: 2023-e-015-r). Informed consent was obtained from all legal guardians of the participants prior to their inclusion in the study.

### 2.2 Participants

A total of 4,404 valid survey responses were obtained for analysis in this research. The inclusion criteria for this study were as follows: (1) participants aged 8–18 years, (2) participants and their legal guardians voluntarily interviewed and signed informed consent (participants signed informed consent by their legal guardians), (3) having the nationality of the People's Republic of China, (4) permanent residents of China (annual travel time not exceeding 1 month), (5) those who can complete the questionnaire by themselves or with the help of a guardian, (6) understanding the meaning of each entry in the questionnaire. The exclusion criteria were: (1) delirious, abnormal spirit, (2) with cognitive impairment, (3) who are participating in similar projects, (4) reluctant collaborators. The study has already been approved. This survey was voluntary and anonymous and the participants had been fully informed of these guidelines.

### 2.3 Data collection

In this study, dedicated survey stations were established in each participating children's hospital. The hospital administrators were responsible for recruiting surveyors. Researchers recruited participants who met the study criteria and personally distributed electronic questionnaires. For participants who had cognitive abilities but needed assistance to complete the questionnaire independently, as well as younger participants who could not fully understand the questionnaire, surveyors provided oral explanations of the questionnaire content as needed and helped participants complete the questionnaire.

### 2.4 Measurements

#### 2.4.1 Three item Loneliness Scale

The assessment of loneliness in this research used the three item Loneliness Scale (T-ILS), developed by Russell (18), is used to rapidly assess an individual's loneliness. The scale consists of 3 items and adopts a 3-level rating (1 = never, 2 = sometimes, 3 = always) with a total score ranging from 3 to 9 points. The higher the total score, the stronger the loneliness. The second question is scored in reverse. Previous research found that T-ILS exhibits high validity and reliability (19). The internal consistency in the present sample was comparable (Cronbach's α = 0.878).

#### 2.4.2 Short form perceived stress scale

In this study, the short form perceived stress scale (PSS-4) was used to quantify perceived stress a self-report questionnaire developed by Cohen et al. (20) that is widely validated and reliable for use in studies of adolescent populations (21). PSS-4 was a validated scale with good internal consistency, and the internal consistency of the present sample is reasonable. The short form perceived stress scale (PSS-4) includes four items, including the sense of loss of control and tension, and adopts a 5-level score (0 = never, 1 = occasionally, 2 = sometimes,3 = often, 4 = always), with a total score of 0 ~ 16. Questions 2 and 3 are scored in reverse, and the higher the score is, the greater the perceived pressure is.

#### 2.4.3 Children's medication adherence

This scale consists of 28 items and six dimensions. The scale adopts Likert 5-point scoring (1 = strongly disagree, 5 = strongly agree), where the dimensions of medication adherence, unsafe medication, and medication difficulty are scored in reverse. The total score of the scale is the sum of the scores of each item, and the higher the score, the higher the medication safety of the subjects. This study selected the dimension of medication adherence for research. In this study, the Cronbach's α coefficient of the scale was 0.913.

### 2.5 Data analysis

Statistical analysis was conducted using SPSS 26.0. The scores of the three scales all follow a normal distribution, expressed as (x ± s), and one-way analysis of variance (ANOVA) was conducted to evaluate the overall intergroup differences in mean scores. Use Pearson correlation analysis to determine the correlation between scores. Perform stratified stepwise regression analysis with medication adherence score as the dependent variable, significant variables in univariate analysis, and PSS-4 and T-ILS scores as independent variables; The mediation effect analysis was conducted with loneliness as the independent variable, medication adherence as the dependent variable, and perceived stress as the mediating variable. The mediation effect test was performed using Model 4 of the SPSS macro program Process developed by Hayes (2017), and the *P*-value of less than 0.05 indicated statistically significant differences. If the 95% confidence interval (95% CI) does not include zero, the effect is considered significant. Using the bootstrap method, we assessed the statistical significance of the mediating effect based on the 95% CI for each path coefficient.

## 3 Result

### 3.1 Participants' sociodemographic characteristics

The final sample comprised of 4,404 participants, of whom 46.93% were female and 53.07% were male. The participants had a mean age of 13.6 years with a standard deviation of 2.85 years. The majority of participants were elementary school students (32.02%), and junior high school students (32.02%). Among all respondents, 68.32% held urban household registration, while 31.68% had rural household registration. Furthermore, 73.8% had resided in urban areas during the past 3 months. A total of 74.6% of participants lived with both parents, whereas 14.2% resided with one parent. Additionally, 85.42% of participants did not have a private room at home. The prevalence of chronic disease among participants was low, at 9.04%. These findings are summarized in Table 1.

**Table 1:** Comparison of perceived stress, loneliness, and medication adherence in 4,404 Chinese children with different demographic characteristics.

Characteristics	*N* (%)	Perceived stress	Loneliness	Medication adherence
Gender				
Male	2,337 (53.07)	6.43 ± 2.76	4.59 ± 1.69	22.72 ± 5.94
Female	2,067 (46.93)	6.68 ± 2.87	4.82 ± 1.70	23.10 ± 5.60
t/F		2.978	4.513	2.162
*P*		0.003^**^	< 0.001^***^	0.031^**^
Age				
< 12	1,069 (24.27)	5.74 ± 2.71	4.39 ± 1.49	22.50 ± 5.85
12~18	3,335 (75.73)	6.81 ± 2.80	7.80 ± 1.75	23.02 ± 5.76
t/F		10.976	6.953	2.552
*P*		< 0.001^***^	< 0.001^***^	0.011^**^
Educational attainment				
Primary school	1,410 (32.02)	5.80 ± 2.79	4.36 ± 1.50	22.74 ± 5.82
Junior high school	1,410 (32.02)	6.55 ± 2.86	4.64 ± 1.73	23.65 ± 5.80
Secondary school	612 (13.89)	7.55 ± 2.36	5.11 ± 1.85	21.77 ± 6.26
High school	852 (19.35)	7.00 ± 2.84	5.00 ± 1.75	22.83 ± 5.19
Junior college	76 (1.73)	6.69 ± 2.43	5.16 ± 1.58	22.28 ± 5.28
Tertiary school	44 (0.99)	6.55 ± 2.82	4.89 ± 1.74	21.66 ± 6.40
t/F		42.005	25.511	10.336
*P*		< 0.001^***^	< 0.001^***^	< 0.001^***^
Chronic diseases				
Yes	398 (9.04)	7.85 ± 2.75	5.55 ± 1.82	22.57 ± 5.28
No	4,006 (90.96)	6.42 ± 2.79	4.61 ± 1.67	22.93 ± 5.83
t/F		−9.808	−10.566	1.190
*P*		< 0.001^***^	< 0.001^***^	0.234
Residence within the past 3 months				
Countryside	1,395 (31.68)	7.06 ± 2.51	4.90 ± 1.75	22.41 ± 6.02
City	3,009 (68.32)	6.31 ± 2.92	4.61 ± 1.67	23.12 ± 5.66
t/F		8.783	5.237	−3.761
*P*		< 0.001^***^	< 0.001^***^	< 0.001^***^
Registered permanent residence				
Countryside	2,256 (51.23)	6.92 ± 2.65	4.83 ± 1.73	22.73 ± 5.94
City	2,148 (48.77)	6.16 ± 2.93	4.57 ± 1.37	23.08 ± 5.62
t/F		8.986	5.076	−2.017
*P*		< 0.001^***^	< 0.001^***^	0.044^**^
Coresiding with others				
Not coresiding with parents	494 (11.2)	7.47 ± 2.37	5.21 ±1.86	21.69 ± 6.26
Coresiding with parents	3,285 (74.6)	6.32 ± 2.85	4.58 ± 1.65	23.04 ± 5.72
Coresiding with father or mother	625 (14.2)	6.99 ± 2.78	4.91 ± 1.73	23.08 ± 5.61
t/F		45.592	35.972	12.169
*P*		< 0.001^***^	< 0.001^***^	< 0.001^***^
Only child				
Yes	3,007 (68.28)	6.69 ± 2.78	4.75 ± 1.70	22.95 ± 5.75
No	1,397 (31.72)	6.25 ± 2.86	4.58 ± 1.69	22.79 ± 5.87
t/F		−4.764	−3.105	−0.850
*P*		< 0.001^***^	0.002^**^	0.395
Time spent with family				
≤ 12 h	2,340 (53.1)	6.82 ± 2.77	4.85 ± 1.75	22.45 ± 5.78
>12 h	2,064 (46.9)	6.23 ± 2.83	4.53 ± 1.63	23.41 ± 5.75
t/F		6.976	6.140	−5.513
*P*		< 0.001^***^	< 0.001^***^	< 0.001^***^
Cohabitants on long-term medications				
Yes	947 (21.50)	6.98 ± 2.87	5.03 ± 1.73	22.67 ± 5.36
No	3,457 (78.50)	6.43 ± 2.79	4.61 ± 1.68	22.96 ± 5.90
t/F		−5.385	−6.744	1.462
*P*		< 0.001^***^	< 0.001^***^	0.144
**Cohabitants' employment as healthcare professionals**				
Yes	708 (16.08)	6.03 ± 2.76	4.61 ± 1.72	23.06 ± 5.53
No	3,696 (83.92)	6.65 ± 2.78	4.72 ± 1.70	22.87 ± 5.83
t/F		5.112	1.535	−0.801
*P*		< 0.001^***^	0.125	0.423
**Had a separate room**				
Yes	642 (14.58)	7.00 ± 2.66	5.02 ± 1.80	22.15 ± 6.03
No	3,762 (85.42)	6.47 ± 2.84	4.64 ± 1.68	23.02 ± 5.73
t/F		4.596	5.144	−3.434
*P*		< 0.001^***^	< 0.001^***^	< 0.001^***^

t/F, *t*-statistic in independent samples *t*-test/F-statistic in one-way ANOVA; ^**^P < 0.05, ^***^P < 0.001.

### 3.2 Scores of perceived stress, loneliness and medication adherence

The scores for groups with varying demographic characteristics are shown in Table 1. The mean scores for perceived stress, loneliness, and medication adherence were 6.55 ± 2.82, 4.70 ± 1.70, and 22.90 ± 5.79.

### 3.3 Correlations among loneliness, perceived stress, and medication adherence

The findings of the Pearson correlation analysis are detailed in Table 2. Loneliness demonstrated a negative correlation with medication adherence (*r* = −0.234, *P* < 0.001), and a positive correlation with perceived stress (*r* = 0.454, *P* < 0.001). Meanwhile, the perceived stress was negatively correlated with medication adherence (*r* = −0.197, *P* < 0.001). These correlations suggest moderate to strong relationships among the three variables.

**Table 2:** Correlation analysis of perceived stress, loneliness, and medication adherence in Chinese children (*n* = 4,404).

Variables	Perceived stress	Loneliness	Medication adherence
Perceived stress	1.000		
Loneliness	0.454^**^	1.000	
Medication adherence	−0.234^**^	−0.197^**^	1.000

^**^P < 0.001.

### 3.4 The results of regression and mediation analyses

**Figure 1:**
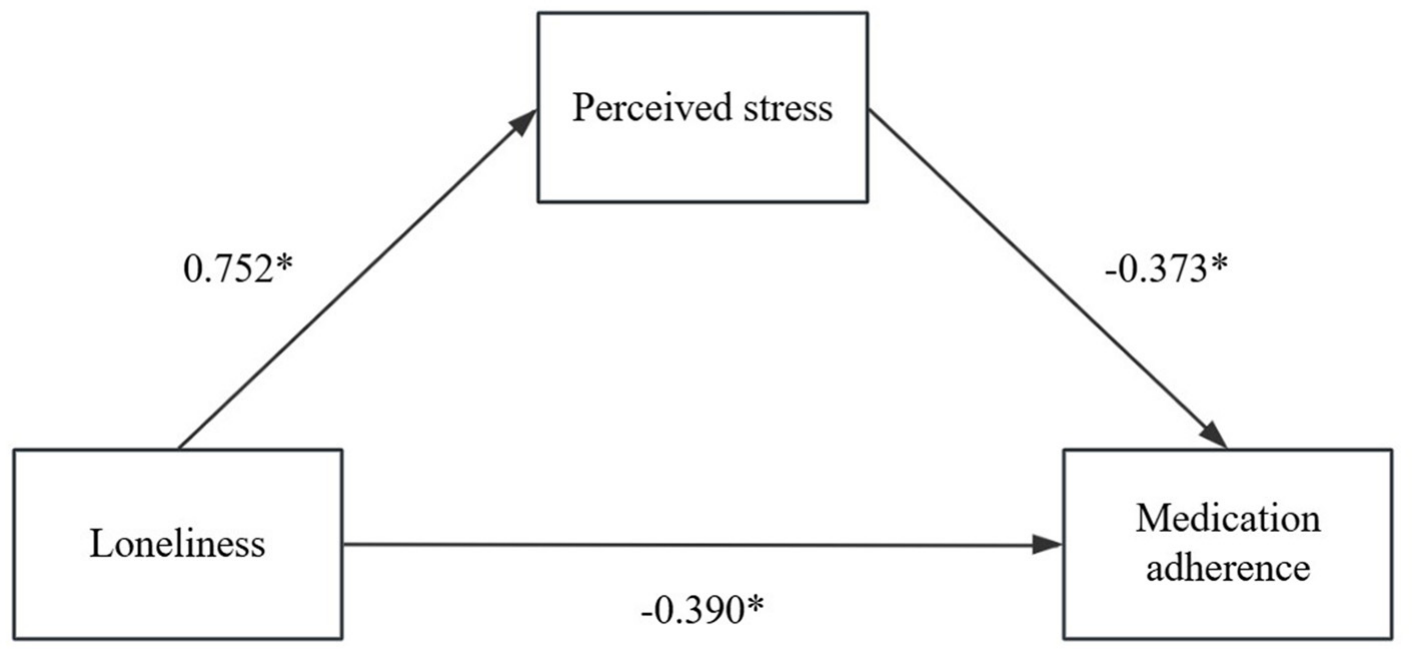
Pathways of influence of the mediating role of perceived stress among loneliness and medication adherence. *p < 0.05.

The correlation analysis indicated the presence of associations among the three factors under investigation. A structural model was developed to examine the mediating effect of perceived stress on the relationship between loneliness and medication adherence as illustrated in Figure 1. The findings presented in Table 3 demonstrate that loneliness is a significant negative predictor of medication adherence (β =-0.670, *P* < 0.001) and a significant positive predictor of perceived stress (β =0.752, *P* < 0.001). Both loneliness and perceived stress were found to negatively predict medication adherence (β = −0.390, *P* < 0.01; β = −0.373, *P* < 0.01, respectively).

**Table 3:** Mediating effect pathway test of perceived stress in Chinese pediatric patients between loneliness and medication adherence.

Independent variable	Dependent variable	Beta (SE)	*t*	*P*	R^2^
Medication adherence	Loneliness	−0.670 (0.050)^a^	−13.340	< 0.001^***^	0.197
Perceived stress	Loneliness	0.752 (0.022)	33.828	< 0.001^***^	0.206
Medication adherence	Loneliness	−0.390 (0.056)	−7.007	< 0.001^***^	0.255
	Perceived stress	−0.373 (0.034)	−11.098	< 0.001^***^	

a This value represents the total effect of loneliness on medication adherence. ***P < 0.001.

In addition, our research used the deviation-corrected percentile bootstrap method to test 95% CI of each path coefficient. The findings regarding the mediation effect are shown in Table 4. The direct effect of loneliness on medication adherence among Chinese children was −0.390 (95% CI: −0.499 to −0.281). The mediating effect was calculated to be −0.280 (95% CI: −0.331 to −0.229), accounting for 41.8% of the total effect. The results showed that the 95% confidence intervals did not include zero, suggesting that perceived stress serves as a partial mediating role.

**Table 4:** Mediating effect test of perceived stress in Chinese pediatric patients between loneliness and medication adherence.

Effect type	Standardized effect size	SE	95% CI		Ratio of effect
			Lower	Upper	
Total effect	−0.670	0.050	−0.769	−0.572	–
Direct effect	−0.390	0.056	−0.499	−0.281	58.2%
Indirect effect	−0.280	0.026	−0.331	−0.229	41.8%

## 4 Discussion

Although previous studies have explored the relationship between psychological factors and medication adherence, this study is the first to confirm that perceived stress mediates the association between loneliness and medication adherence among children, thereby extending the applicability of relevant theoretical models. The findings reveal a negative association between loneliness and medication adherence (β = −0.670, *P* < 0.001), with perceived stress also demonstrated a significant correlation with adherence behaviors (β = −0.373, *P* < 0.001). The results suggest that perceived stress may partially mediate the relationship between loneliness and medication adherence, with the mediating effect accounting for 41.8% (95% CI: −0.331 to −0.229) of the association. This indicates a potential interconnection between loneliness and medication adherence; wherein perceived stress may act as a partial mediator. Notably, this is the first study to demonstrate that loneliness can affect medication compliance through the mediating effect of perceived stress in Chinese pediatric patients.

Consistent with Hypothesis 1, our study identified a predictive relationship between the degree of loneliness and medication adherence, thereby corroborating previous research (22, 23). Specifically, reduced levels of loneliness were associated with improved adherence to medication regimens, whereas elevated loneliness was significantly linked to increased instances of non-compliance. Among participants with varying demographic characteristics, loneliness consistently emerged as a negative predictor of medication adherence. Improving medication adherence may have a substantial effect on the overall health of the population. This study found no significant difference in loneliness scores between only children and those with siblings. This finding may challenge existing stereotypes, as discussed in previous studies. Shengjie Lin (24) found that Chinese society often assumes only children experience greater loneliness; however, evidence indicates that individuals raised with siblings report higher levels of loneliness.

Hypothesis 2 was substantiated, indicating that loneliness is a predictor of perceived stress. Previous studies have established a relationship between stress and loneliness (25). Furthermore, loneliness may serve as a mediating variable, modulating perceived stress levels. For example, it may partially mediate the relationship between perceived stress and depressive symptoms (26). This study reveals that the loneliness among pediatric patients is a significant predictor of both perceived stress (β = 0.752, *P* < 0.05) and medication adherence (β = −0.390, *P* < 0.05). Meanwhile, Hypothesis 3 has been confirmed, showing that perceived stress can indeed impact medication adherence (β = −0.373, *P* < 0.001). These findings indicate that interventions targeting the reduction of loneliness may effectively decrease perceived stress and improve medication adherence among pediatric patients.

This model is of considerable importance when perceived stress is regarded as a direct mediating variable, thereby supporting our Hypothesis 4. The finding of this study indicated that loneliness exerts a sequential indirect impact on medication adherence via perceived stress. Prior studies have demonstrated that perceived stress can influence medication adherence among patients with hypertension (27). This finding is consistent with our research, which also identifies a negative correlation between perceived stress and medication adherence. The relationship between stress and medication adherence was found to be statistically significant among parents of children with epilepsy (28). Our research is consistent with the principles of triadic reciprocal determinism (29), a theoretical framework that highlights the dynamic interplay among environmental factors (e.g., loneliness), personal factors (e.g., perceived stress), and behavioral factors (e.g., medication adherence). Increased levels of perceived stress in pediatric patients re correlated with reduced adherence to medication regimens, subsequently intensifying experiences of loneliness.

This study is of considerable significance in enhancing medication adherence. Previous research has predominantly focused on physiological determinants of medication adherence; our findings reveal that loneliness can negatively predict medication adherence through the mediating role of perceived stress. These results expand the understanding of psychological pathways that can be leveraged to improve medication adherence. Based on the findings of this study, it is recommended that early screening be implemented for children exhibiting elevated levels of loneliness. Furthermore, the integration of stress management modules into chronic disease management is deemed essential. Notably, therapeutic play has been identified as a particularly effective strategy. Therapeutic games, as conceptualized by Friedman (30), consist of a series of structured activities tailored to children of different ages, cognitive development stages, and conditions. Friedman argues that these games are instrumental in reducing stress and tension in children while concurrently fulfilling therapeutic goals.

The perception and effective utilization of social support are crucial for maintaining the mental health of children, particularly those who are sensitive and receive inadequate social support. Primarily, this support is derived from parental figures. Given the limited cognitive development in pediatric patients, it is imperative to implement a family-centered communication model. Medical personnel engage in communication with caregivers to assist parents in comprehending and managing the present condition of the illness (31). This interaction serves to mitigate the tension, pain, and anxiety associated with the disease, while also enhancing the communication skills of family members. Moreover, both online and offline patient communities offer a platform for parents to share experiences, thereby supporting pediatric patients in enhancing their psychological wellbeing and ultimately improving treatment adherence.

The findings of this study hold significant clinical implications for optimizing pediatric medication management in China. Firstly, the pressure and loneliness experienced by children can predict medication-related behaviors, particularly among those with chronic diseases, where poor medication adherence may lead to adverse health outcomes. Therefore, implementing loneliness and stress screenings in hospitals allows for earlier intervention, which is essential to improve medication adherence. Secondly, family involvement in medication management is critical, especially for children with chronic diseases requiring long-term treatment. In Chinese families, where caregivers often play a central yet passive role, establishing family-supported psychological interventions, such as play therapy and communication training, can enhance both caregiving capacity and adherence. In addition, Shared decision-making involving both clinicians and patients' families is receiving growing emphasis. In Chinese healthcare context, however, constrained physician-patient communication time frequently relegates caregivers to a passive role in the decision-making process. Promoting family-centered communication within time-limited clinical encounters can facilitate shared decision-making, thereby enhance medication adherence and improve health outcomes.

Although the basic demographic variables such as age and gender are controlled in this study, there are still unmeasured confounding factors. Primarily, the cross-sectional design constrains our capacity to establish definitive causal relationships among loneliness, medication adherence, and perceived stress. To elucidate these temporal relationships, future research employing longitudinal designs is recommended. Second, while perceived stress serves as a partial mediator in the relationship between loneliness and medication adherence, it does not entirely explain this association. Future research should incorporate additional variables into the analytical model to provide a more comprehensive understanding. Moreover, the study did not account for the contribution of family function, which is particularly important. Conflicts caused by differences in parental care between grandparents and parents may affect children's psychological status. Finally, data collection relied solely on standardized self-report questionnaires, a method that may introduce reporting biases. Integrating qualitative methodologies, such as in-depth interviews, could enhance the validity and robustness of the findings.

This study substantiates the role of perceived stress as a critical mechanism in the transformation of loneliness into poor adherence behaviors among Chinese children., suggesting that integrating psychosocial intervention can optimize the management of pediatric chronic diseases.

## 5 Conclusion

This study offers empirical evidence elucidating the pathways connecting loneliness to medication adherence. Loneliness is associated with medication adherence both directly and indirectly. Specifically, it diminishes medication adherence among pediatric patients through the mediating effect of perceived stress. This research highlights the pivotal role of psychological factors in understanding the mechanisms underlying the relationship between loneliness and medication adherence. Intervention strategies aimed at enhancing medication adherence and promoting healthy behaviors may benefit from approaches that mitigate loneliness, reduce perceived stress, address stressors, prevent tension, and enhance perceptual control. Future longitudinal studies are warranted to further explore and clarify the potential pathways linking loneliness to medication adherence.

## Data Availability

The original contributions presented in the study are included in the article/supplementary material, further inquiries can be directed to the corresponding author.
